# Fibroblast growth factor pathway promotes glycolysis by activating LDHA and suppressing LDHB in a STAT1-dependent manner in prostate cancer

**DOI:** 10.1186/s12967-024-05193-9

**Published:** 2024-05-19

**Authors:** Yongkang Ye, Fukan Yang, Zhanhao Gu, Wenxuan Li, Yinjiao Yuan, Shaoqian Liu, Le Zhou, Bo Han, Ruinian Zheng, Zhengguo Cao

**Affiliations:** 1 Department of Urology, The Tenth Affiliated Hospital of Southern Medical University (Dongguan people’s hospital), 523059 Dongguan, China; 2 Department of Urology, Guangdong Medical University, Graduate School, 524002 Zhanjiang, China; 3 Department of Oncology, Dongguan Institute of Clinical Cancer Research, Dongguan Key Laboratory of Precision Diagnosis and Treatment for Tumors, The Tenth Affiliated Hospital of Southern Medical University (Dongguan people’s hospital), 523059 Dongguan, China; 4 The First School of Clinical Medicine, Southern Medical University, 510510 Guangzhou, China

**Keywords:** Prostate cancer, Fibroblast growth factor, LDH, Glycolysis, Pathway

## Abstract

**Background:**

The initiation of fibroblast growth factor 1 (FGF1) expression coincident with the decrease of FGF2 expression is a well-documented event in prostate cancer (PCa) progression. Lactate dehydrogenase A (LDHA) and LDHB are essential metabolic products that promote tumor growth. However, the relationship between FGF1/FGF2 and LDHA/B-mediated glycolysis in PCa progression is not reported. Thus, we aimed to explore whether FGF1/2 could regulate LDHA and LDHB to promote glycolysis and explored the involved signaling pathway in PCa progression.

**Methods:**

In vitro studies used RT‒qPCR, Western blot, CCK-8 assays, and flow cytometry to analyze gene and protein expression, cell viability, apoptosis, and cell cycle in PCa cell lines. Glycolysis was assessed by measuring glucose consumption, lactate production, and extracellular acidification rate (ECAR). For *in vivo studies*, a xenograft mouse model of PCa was established and treated with an FGF pathway inhibitor, and tumor growth was monitored.

**Results:**

FGF1, FGF2, and LDHA were expressed at high levels in PCa cells, while LDHB expression was low. FGF1/2 positively modulated LDHA and negatively modulated LDHB in PCa cells. The depletion of FGF1, FGF2, or LDHA reduced cell proliferation, induced cell cycle arrest, and inhibited glycolysis. LDHB overexpression showed similar inhibitory effect on PCa cells. Mechanistically, we found that FGF1/2 positively regulated STAT1 and STAT1 transcriptionally activated LDHA expression while suppressed LDHB expression. Furthermore, the treatment of an FGF pathway inhibitor suppressed PCa tumor growth in mice.

**Conclusion:**

The FGF pathway facilitates glycolysis by activating LDHA and suppressing LDHB in a STAT1-dependent manner in PCa.

**Supplementary Information:**

The online version contains supplementary material available at 10.1186/s12967-024-05193-9.

## Introduction

Prostate cancer (PCa) is a malignant tumor with the highest incidence rate among men [1]. The specific symptoms of early PCa are not obvious, which brings challenges to the accurate diagnosis [2]. PCa patients with obvious symptoms are often diagnosed at a late stage and thus miss the optimal treatment opportunity, leading to increased mortality [3]. The current conventional treatment methods include radical resection surgery, radiation therapy, chemotherapy, and endocrine therapy [4]. However, most patients who are initially sensitive to endocrine therapy develop resistance to treatment in the later stages and progress to castration-resistant prostate cancer (CRPC), increasing the difficulty of treatment [5]. Therefore, understanding the potential mechanisms that promote the development of PCa remains a top priority.

Changes in glucose metabolism are one of the hallmarks of cancer development [6]. Aerobic glycolysis (the Warburg effect) refers to the process by which normal cells extract energy from glucose mainly through oxidative phosphorylation without a lack of oxygen, while tumor cells metabolize more glucose into lactate [7]. Lactate is the end product of glycolysis and can promote tumor growth and participate in tumor metabolic symbiosis [8]. In the Warburg effect, 85% of pyruvate is converted to lactate by lactate dehydrogenase (LDH) [9], so LDH is considered a key metabolic enzyme. LDH consists of LDHA and LDHB in tetrameric form. LDHA has a high affinity for pyruvate, while LDHB has a high affinity for lactate [8, 10]. LDHA preferentially reduces pyruvate to lactate, whereas LDHB favors the reverse reaction [11]. LDHA overexpression has been detected in many cancers [12]. This leads to excessive lactate accumulation in cancer cells, thus increasing acidification of the tumor microenvironment and promoting invasion, metastasis and angiogenesis [13]. LDHA is considered a key enzyme involved in cancer metabolism and can serve as a prognostic indicator [13]. A high level of LDH is closely related to a poor prognosis in patients with ovarian cancer [14]. LDHA is reported to facilitate glycolysis, growth, and metastasis in thyroid cancer [15]. Moreover, LDHA accelerates cell proliferation, migration, invasion, and glycolysis in PCa [16]. Compared to the significant carcinogenic effect of LDHA in most cancers, the association between LDHB and tumors is much more complex [17]. Studies have confirmed that LDHB can be silenced by methylation in some cancers, whereas LDHB is also highly expressed in other cancers [8]. LDHB is silenced by hypermethylation in PCa [18]. Liu et al. suggested that LDHA upregulation or LDHB downregulation can enhance tumor growth in PCa [19]. However, the specific mechanism through which LDHA and LDHB regulate cancer metabolism has not been fully elucidated, and their impact on PCa glycolysis requires further research.

Growth factors play critical roles in maintaining normal cellular development and homeostasis in human prostate [20]. Fibroblast growth factor (FGF) is a large family of growth factors that exert their biological functions by binding to their receptors (FGFRs) [21]. They regulate the basic development pathways of multiple organ systems and many pathological processes, including cell proliferation, migration, and angiogenesis [22]. The four types of FGFRs are FGFR1, FGFR2, FGFR3, and FGFR4. Various FGFs bind to different FGFRs to varying degrees, with FGF1 and FGF2 being ligands for all different FGFRs [23]. The abnormal activation of FGFs and FGFRs is a pathological condition that contributes to the occurrence of various cancers [24], including PCa [25], breast cancer [26], and lung cancer [27]. The expression of FGF1, FGF2, FGF7, FGF9, and FGF10 has been detected in prostate stromal fibroblasts [28]. Currently, most related research has focused on FGF1 and FGF2, which are widely expressed in various cells [29]. FGF1 and FGF2 are believed to play vital roles in stimulating endothelial cell proliferation and promoting angiogenesis [30]. FGF1 promotes cell proliferation, migration, and invasion in different cancers, such as ovarian cancer [31], oral squamous cell carcinoma [32], and colorectal cancer [33]. FGF1 can induce the expression of matrix metalloproteinases in PCa cells, thus participating in malignant development [34]. FGF2 can directly stimulate angiogenesis and play a biological role in cancer by inducing cell migration, proliferation, and differentiation [35, 36]. Clinical studies have shown that high serum levels of FGF2 can serve as an effective predictor of poor prognosis in patients with hepatocellular carcinoma [37]. Similarly, elevated serum levels of FGF2 are also detected in PCa patients [38, 39]. FGF2 overexpression has been confirmed to promote PCa cell proliferation and angiogenesis [38]. In addition, substantial literature has reported the involvement of the FGF signaling pathway in systemic metabolic regulation [40]. For example, FGF21 inhibits metabolic disorders and promotes metabolic homeostasis in the liver [41]. FGF7 promotes glucose production, lactate production, and ATP production in breast cancer [42]. However, the specific mechanism through which FGF1 and FGF2 regulate aerobic glycolysis in prostate cancer remains unclear.

The objective of this study was to explore the specific molecular mechanism by which FGF1/2 regulates LDHA and LDHB to promote PCa glycolysis and to study the influence of the FGF-LDHA/LDHB metabolic pathway on PCa development.

## Materials and methods

### Bioinformatics analysis

TcoFbase database (http://tcof.liclab.net/TcoFbase/search.php) was utilized to predict the downstream targets of FGF1 and FGF2. GEPIA (http://gepia2.cancer-pku.cn/#index) database was exploited to determine the expression of LDHA, LDHB, FGF1 and FGF2 in normal tissues and PCa tissues. UALCAN (https://ualcan.path.uab.edu/index.html) database was used to illustrate the relationship between LDHA expression and the survival of prostate adenocarcinoma (PRAD) patients. THE HUMAN PROTEIN ATLAS (HPA) database (https://www.proteinatlas.org/) was used to explore the protein expression of LDHA and LDHB in prostate adenocarcinoma samples and normal samples. The FGF pathway related genes were predicted using the PathCards database (https://pathcards.genecards.org/Card/fgf_signaling_pathway?queryString=FGF). The JASPAR database (https://jaspar.genereg.net/) was used to predict the binding sites between STAT1 and LDHA or LDHB. The expression of STAT1 in PRAD was analyzed using the TCGA data downloaded from the UCSC Xena database (https://xena.ucsc.edu/).

### Cell culture

Four PCa cell lines (LNCaP, PC3, 22RV1, and C4-2) and one normal prostate epithelial cell line (RWPE-2) were purchased from the Cell Bank of the Chinese Academy of Sciences (Shanghai, China). The cells were incubated in RPMI-1640 medium (Gibco, USA) supplemented with 10% FBS at 37 °C with 5% CO_2_.

### Cell transfection

To silence FGF1, FGF2, or LDHA, PC3 and 22RV1 cells were transfected with specific short hairpin RNAs (shRNAs), sh-FGF1, sh-FGF2, sh-LDHA as well as with each corresponding negative control (NC, sh-NC). Furthermore, the full-length LDHB or STAT1 sequence was inserted into the pcDNA3.1 vector (Geenseed Biotech, China) to overexpress LDHB or STAT1 in cells. All the plasmids and vectors were purchased from GenePharma (Shanghai, China) and transfected into cells using Lipofectamine 3000 (Invitrogen, USA) for 48 h. Thereafter, the cells were harvested for the following assays.

### RT‒qPCR

Total RNA was extracted from PC3 and 22RV1 cells by TRIzol reagent (Invitrogen). Next, the isolated RNA was reverse transcribed to cDNA by the PrimeScript™ One Step RT‒PCR Kit (Takara, Japan). Then, qPCR analysis was conducted with SYBR Green PCR Master Mix (Applied Biosystems, USA) on a StepOnePlus Real-Time PCR System (Applied Biosystems). Gene expression was detected utilizing the 2^−ΔΔCT^ method and was normalized to that of GAPDH. The sequences of primers are shown in Table 1. Each biological samples were run in triplicate.

**Table 1:** Primer sequences used in RT‒qPCR

Genes	Sequences
FGF1	F: 5’-TATACGGCTCACAGACACC-3’
	R: 5’-TCTCTGCATGCTTCTTGGA-3’
FGF2	F: 5’-TGTGTCTATCAAAGGAGTGTG-3’
	R:5’-CGTAACACATTTAGAAGCCAG-3’
LDHA	F: 5’-TTCCAGTGTGCCTGTATGG-3’
	R: 5’-TTATCAGTCCCTAAATCTGGGTG-3’
LDHB	F: 5’-ACAATAAGATCACTGTAGTGGG-3’
	R: 5’-CATCAGCCAGAGACTTTCC-3’
GAPDH	F: 5’-TCAAGATCATCAGCAATGCC-3’
	R: 5’-CGATACCAAAGTTGTCATGGA-3’

### Western blot

Total proteins were extracted from PC3 and 22RV1 cells with RIPA buffer (Beyotime, China). Protein samples were separated via 12% SDS‒PAGE and subsequently transferred to PVDF membranes (Millipore, USA). The PVDF membranes were blocked with 5% nonfat milk and then probed with the following primary antibodies: anti-LDHA (#2012, 1:1000; Cell Signaling Technology, Danvers, MA); anti-LDHB (ab85319, 1:1000; Abcam, Cambridge, UK); anti-STAT1 (ab234400, 1:1000; Abcam); anti-FGF1 (ab207321, 1:1000; Abcam); anti-FGF2 (ab208687, 1:1000; Abcam); and anti-GAPDH (ab9485, 1:2500; Abcam) overnight at 4 °C. Afterwards, the sections were incubated with secondary antibodies (Abcam, ab7090, 1:2000). The protein bands were detected with an enhanced chemiluminescence (ECL) kit (Millipore) and quantified using ImageJ software.

### CCK-8 assay

**A** CCK-8 kit (CK-04, Dojindo, Japan) was used to test cell viability. The PC3 and 22RV1 cells (1 × 10^4^) were seeded into 96-well plates and cultured for 24 h. Next, 10 µL of CCK-8 solution was added to each well and incubated for 2 h. The absorbance at 450 nm was measured with a microplate reader (Molecular Devices, USA).

### Flow cytometry analysis

For cell apoptosis detection, a FITC-Annexin V Apoptosis Detection Kit (BD Biosciences, San Jose, CA) was used according to the manufacturer’s instructions. Cells were rinsed with cold PBS and resuspended in 1 × binding buffer. Next, cells were dyed with 5 µl of Annexin V-FITC and 5 µl of propidium iodide in the dark at room temperature (RT) for 15 min. For cell cycle detection, cells were rinsed with cold PBS and fixed in 70% cold ethanol at 4 °C overnight. Afterwards, the rinsed cells were resuspended in a 500 µl mixture comprising 50 µg/ml propidium iodide and an equivalent volume of RNase A in the dark at RT for 30 min. A FACS Caliber (Beckman Coulter, USA) was used for acquiring the cells. FlowJo_V10 software was used for apoptosis analysis, and ModFit LT software was used for cycle analysis.

### Glucose consumption detection

A glucose uptake colorimetric assay kit (Sigma Aldrich, USA) was used to detect glucose consumption in the cells. PC3 and 22RV1 cells (2 × 10^3^) were seeded into a 96-well plate and starved of glucose by pre-culturing with 100 µl of KRPH buffer containing 2% BSA. After 30 min, 10 µl of 2-DG was added, and the mixture was cultured for 20 min at RT. A microplate reader (Molecular Devices) was used to measure the absorbance at 412 nm.

### Lactate production detection

PC3 and 22RV1 cells (2 × 10^3^) were seeded into a 96-well plate for 6 h. Next, the culture medium was collected, and 0.5 ml of KRPH was applied for dilution. A lactate colorimetric assay kit was used to measure the lactate concentration in accordance with the manufacturer’s instructions. A microplate reader was used to measure the absorbance at 450 nm, after which the absorbance was normalized to the protein concentration.

### Detection of the extracellular acidification rate (ECAR)

An XF96 flux analyzer (Seahorse Bioscience, USA) was used to detect the ECAR. The PC3 and 22RV1 cells (1 × 10^4^) were grown in an XF 96-well plate overnight. The ECAR was detected in media supplemented with glucose (10 mM), oligomycin (1 µM), and 2-DG (50 mM).

### Luciferase reporter assay

The binding sites between STAT1 and the LDHA or LDHB promoter were obtained from the JASPAR database. The wild-type (WT) or mutated (Mut) STAT1 binding sites to the LDHA or LDHB promoter were inserted into the pGL3 luciferase reporter vector (Promega, USA) to generate pGL3-LDHA promoter-Wt/Mut or pGL3-LDHB promoter-Wt/Mut, respectively. The cells were co-transfected with pGL3-LDHA/LDHB promoter-Wt/Mut and pcDNA3.1-STAT1 or NC for 48 h. The Luciferase Reporter Assay System (Promega) was used to determine luciferase activity.

### ChIP assay

Cells were cross-linked with 1% formaldehyde for 10 min at RT. Then, cells were treated with lysis buffer, and the chromatin was sheared to DNA fragments by sonication. Chromatin was centrifuged for 15 min, after which the supernatant was diluted with dilution buffer. The supernatant was cultured with magnetic beads (Millipore) and an anti-STAT1 antibody (Abcam) overnight at 4 °C. Next, the samples were washed, and ChIP DNA was subjected to elution for 10 min. Proteinase K was utilized to incubate with the supernatant. After purification, the extracted DNA was subjected to amplification via qPCR analysis.

### Tumor xenograft models

Ten male BALB/c nude mice (4–6 weeks; 16–20 g) were purchased from the Vital River (Beijing, China). The protocol of animal experiments was approved by the Ethics Committee of The Tenth Affiliated Hospital of Southern Medical University (certificate number: WTLH2020041517). Mice were randomized into NC and LY2874455 groups (*n* = 5 per group). PC3 cells (5 × 10^6^) were subcutaneously injected into the axillary area of the mice. For LY2874455 treatment (an FGFR inhibitor), mice received 3 mg/kg LY2874455 (ab216313, Abcam) orally for seven days. Mice in the NC group received no treatment. Tumor size was measured using Vernier calipers and recorded every seven days. After thirty-five days, mice were sacrificed by cervical dislocation, and the tumors were dissected, photographed, and weighed.

Mice received intraperitoneal injection of 2.5 mg/100 µL of XenoLight D-luciferin for 10 min. Bioluminescence images were obtained with an IVIS 100 imaging system (PerkinElmer).

### Statistical analysis

The statistical data were analyzed using GraphPad Prism 8.0 and are presented as the means ± SD of three individual repeats. The data were analyzed by Student’s t test or one-way ANOVA followed by Bonferroni post hoc test. *p* < 0.05 indicated statistical significance.

## Results

### LDHA and LDHB are differentially expressed in PCa and are correlated with FGF1/2 expression

**Fig. 1:**
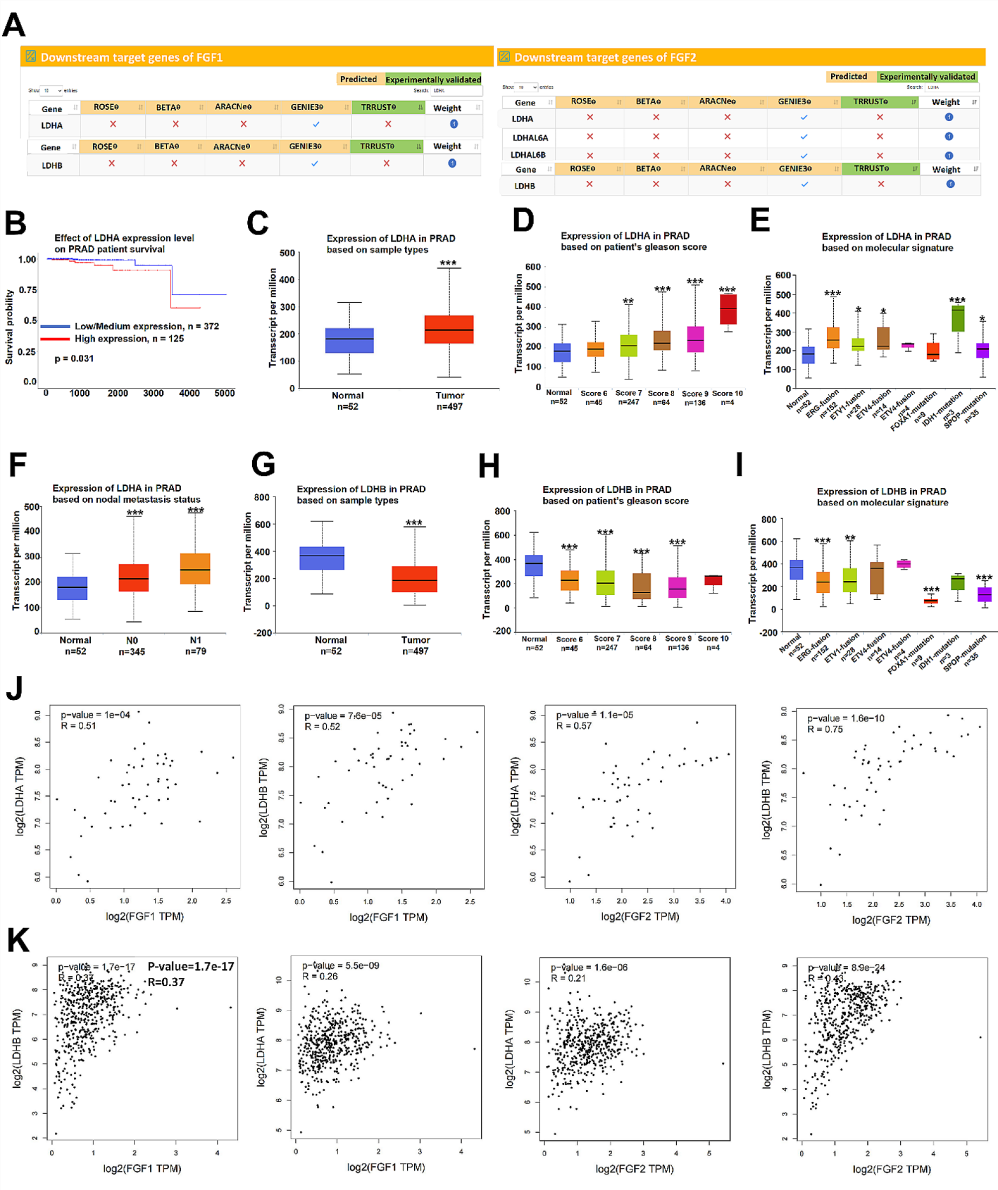
LDHA and LDHB are differentially expressed in PCa and are correlated with FGF1/2 expression. (**A**) The TcoFbase database was utilized to predict the downstream targets of FGF1/2. The prediction outcomes showed that LDHA and LDHB can be targeted by FGF1/2. (**B**) UALCAN analysis of the effect of LDHA expression on PRAD patient survival (based on the TCGA dataset). (**C-F**) UALCAN analysis of LDHA expression in PRAD patients stratified according to sample type, Gleason score, molecular signature, and nodal metastasis status. (**G-I**) UALCAN analysis of LDHB expression in PRAD patients stratified according to sample type, Gleason score, and molecular signature. (**J-K**) Spearman correlation analysis for FGF1/2 and LDHA/LDHB in normal prostate tissues (**j**) and prostate adenocarcinoma (PRAD) tissues (**k**). ^*^p < 0.05, ^**^p < 0.01, ^***^p < 0.001

According to the TcoFbase database (http://tcof.liclab.net/TcoFbase/search.php), the downstream target genes of FGF1/2 were predicted through five different prediction methods (ROSE, BETA, GENIE3, ARACNe and TRRUST). LDHA and LDHB were predicted as downstream targets of FGF1 and FGF2 based on the GENIE3 method (Fig. 1A). UALCAN (https://ualcan.path.uab.edu/index.html) analysis of PCa-related data in the TCGA database illustrated the relationship between LDHA expression and the survival of prostate adenocarcinoma (PRAD) patients as well as the LDHA expression pattern in the PRAD cohort based on sample type, patient Gleason score, molecular signature, and nodal metastasis status. The outcomes showed that high LDHA expression predicted a decreased survival probability in PRAD patients, and low LDHA expression predicted an increased survival probability (Fig. 1B). LDHA was strongly expressed in tumor tissues compared to normal tissues at mRNA (Fig. 1C) and protein levels (Fig. S1A). With increasing Gleason score, the expression of LDHA tended to increase (Fig. 1D). Furthermore, in comparison with that in normal controls, LDHA expression in molecular signatures was upregulated (Fig. 1E). In addition, LDHA expression in N1 and N0 tumors was greater in patients with nodal metastasis than in normal controls (Fig. 1F). In addition, the UALCAN database also showed that LDHB expression was lower in PCa tissues and in patients with different Gleason scores and molecular signatures (Fig. 1G-I). Similarly, the LDHB protein was lowly expressed in PCa tissues compared with normal samples (Fig. S1B). Moreover, based on the GEPIA (http://gepia2.cancer-pku.cn/#index) database, we used Spearman correlation analysis to verify the correlation between LDHA/LDHB and FGF1/FGF2 in normal tissues and PCa tissues. We found that these genes were significantly correlated only in PCa tumor tissues but not in normal tissues (Fig. 1J-K). Overall, we confirmed that LDHA is strongly expressed while LDHB is weakly expressed in PCa and their expression is correlated with FGF1/2 expression.

### FGF1/2 positively regulates LDHA and negatively regulates LDHB

**Fig. 2:**
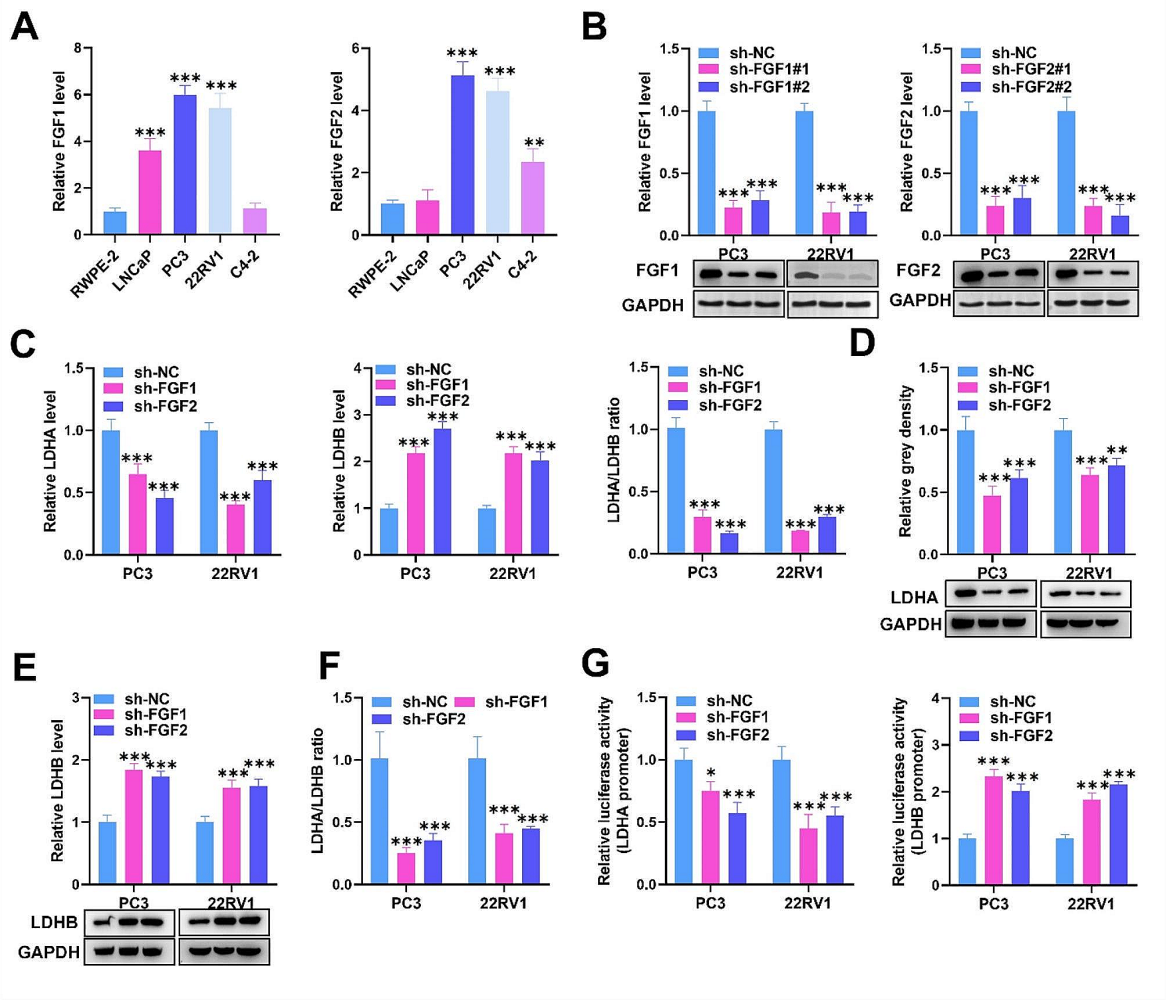
FGF1/2 positively regulates LDHA and negatively regulates LDHB. (**A**) RT‒qPCR analysis of FGF1 or FGF2 in PCa cells. (**B**) RT‒qPCR and western blot analysis of the transfection efficiency of sh-FGF1 or sh-FGF2 in PC3 and 22RV1 cells. (**C-F**) RT‒qPCR and western blot analysis of LDHA expression, LDHB expression, and the LDHA/LDHB ratio in PC3 and 22RV1 cells after transfection with sh-FGF1 or sh-FGF2. (**G**) A luciferase reporter assay was implemented to test the impact of FGF1/2 depletion on the luciferase activity of the LDHA promoter or LDHB promoter. The data is representative of at least three independent experiments. ^*^p < 0.05, ^**^p < 0.01, ^***^p < 0.001

The specific regulatory relationship between FGF1/FGF2 and LDHA/LDHB in PCa was further explored. We first validated the expression of FGF1 and FGF2 in PCa cells and the normal cell line RWPE-2. RT–qPCR revealed that FGF1 was overexpressed in LNCaP, PC3, and 22RV1 cells and that FGF1 was overexpressed in PC3, 22RV1, and C4-2 cells (Fig. 2A). Therefore, we selected PC3 and 22RV1 cells for subsequent assays. To establish FGF1/2-silenced cell lines, we transfected sh-FGF1 or sh-FGF2 plasmids into PC3 and 22RV1 cells. RT‒qPCR and western blot confirmed that both sh-FGF1#1 and sh-FGF1#2 significantly decreased FGF1 expression in cells. Similarly, sh-FGF2#1 or sh-FGF2#2 transfection suppressed FGF2 expression (Fig. 2B). Next, we explored the impacts of FGF1/2 silencing on LDHA/LDHB expression in PC3 and 22RV1 cells. We found that FGF1/2 depletion notably decreased LDHA mRNA and protein levels while increasing LDHB mRNA and protein levels (Fig. 2C-E). Moreover, the LDHA/LDHB ratio was inhibited by FGF1/2 silencing (Fig. 2C**&F**). Furthermore, luciferase reporter assays verified that FGF1/2 deficiency notably reduced LDHA promoter activity while increasing LDHB promoter activity (Fig. 2G). In brief, we confirmed that FGF1/2 positively regulates LDHA and negatively regulates LDHB in PCa.

### FGF1/2 regulates the apoptosis, cell cycle arrest, and glycolysis of PCa cells

**Fig. 3:**
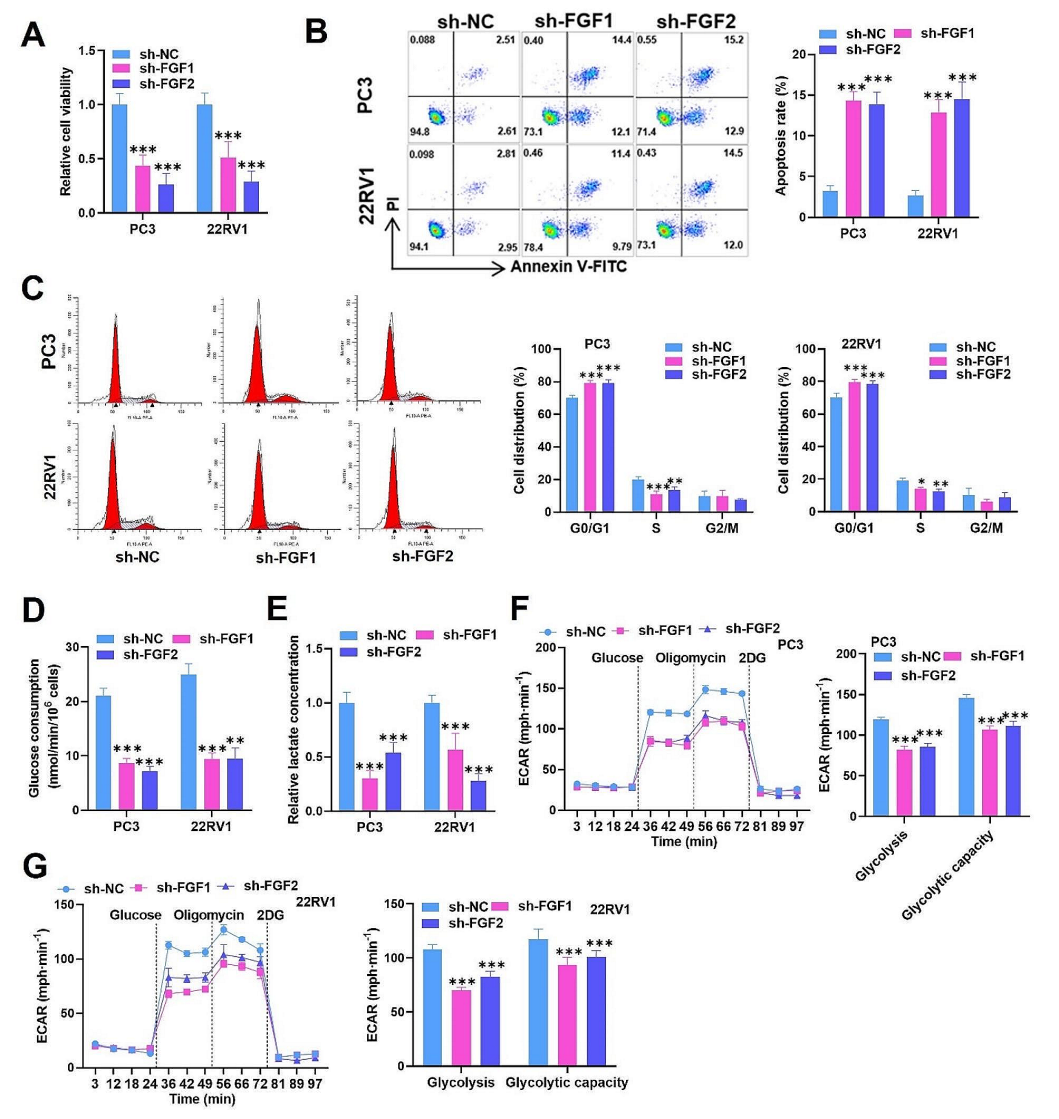
FGF1/2 regulates apoptosis, cell cycle arrest, and glycolysis in PCa cells. (**A**) PC3 and 22RV1 cell viability was tested utilizing a CCK-8 kit in the sh-NC, sh-FGF1, and sh-FGF2 groups. (**B-C**) Cell apoptosis and cell cycle progression were assessed via flow cytometry in different cell groups. (**D-E**) Glucose consumption and lactate concentration were determined in different cell groups. (**F-G**) The ECAR test also confirmed the glycolytic and glycolytic capacity of FGF1/2 in different cell lines. The data is representative of at least three independent experiments. ^*^p < 0.05, ^**^p < 0.01, ^***^p < 0.001

In the next step, we wanted to explore the biological functions of FGF1/2 in PCa cells. For this purpose, functional assays were conducted. Through CCK-8 assay, we found that FGF1/2 knockdown markedly restrained the viability of PC3 and 22RV1 cells relative to that of control cells (Fig. 3A). The cell apoptotic rate was measured using flow cytometry, and we demonstrated that the increase in the apoptotic ratio of PC3 and 22RV1 cells was caused by FGF1/2 knockdown (Fig. 3B). Additionally, flow cytometry further showed that, in comparison with that in control cells, FGF1/2 depletion resulted in cell cycle arrest at the G0/G1 phase in cells, and the S-phase ratio was reduced by FGF1/2 depletion (Fig. 3C). The proliferation of tumor cells is frequently accompanied by metabolic alterations. Thus, we tested the impacts of FGF1/2 depletion on glycolytic metabolism. Our results showed that FGF1/2 deficiency notably reduced glucose consumption and lactate production (Fig. 3D-E). Furthermore, the ECAR test also proved that glycolysis and the glycolytic capacity could be reduced by FGF1/2 deficiency in PC3 and 22RV1 cells (Fig. 3F-G). Thus, we confirmed that FGF1/2 facilitates cell proliferation, cell cycle progression, and glycolysis but suppresses apoptosis in PCa cells.

### LDHA silencing increases apoptosis, induces cell cycle arrest, and suppresses glycolysis in PCa cells

**Fig. 4:**
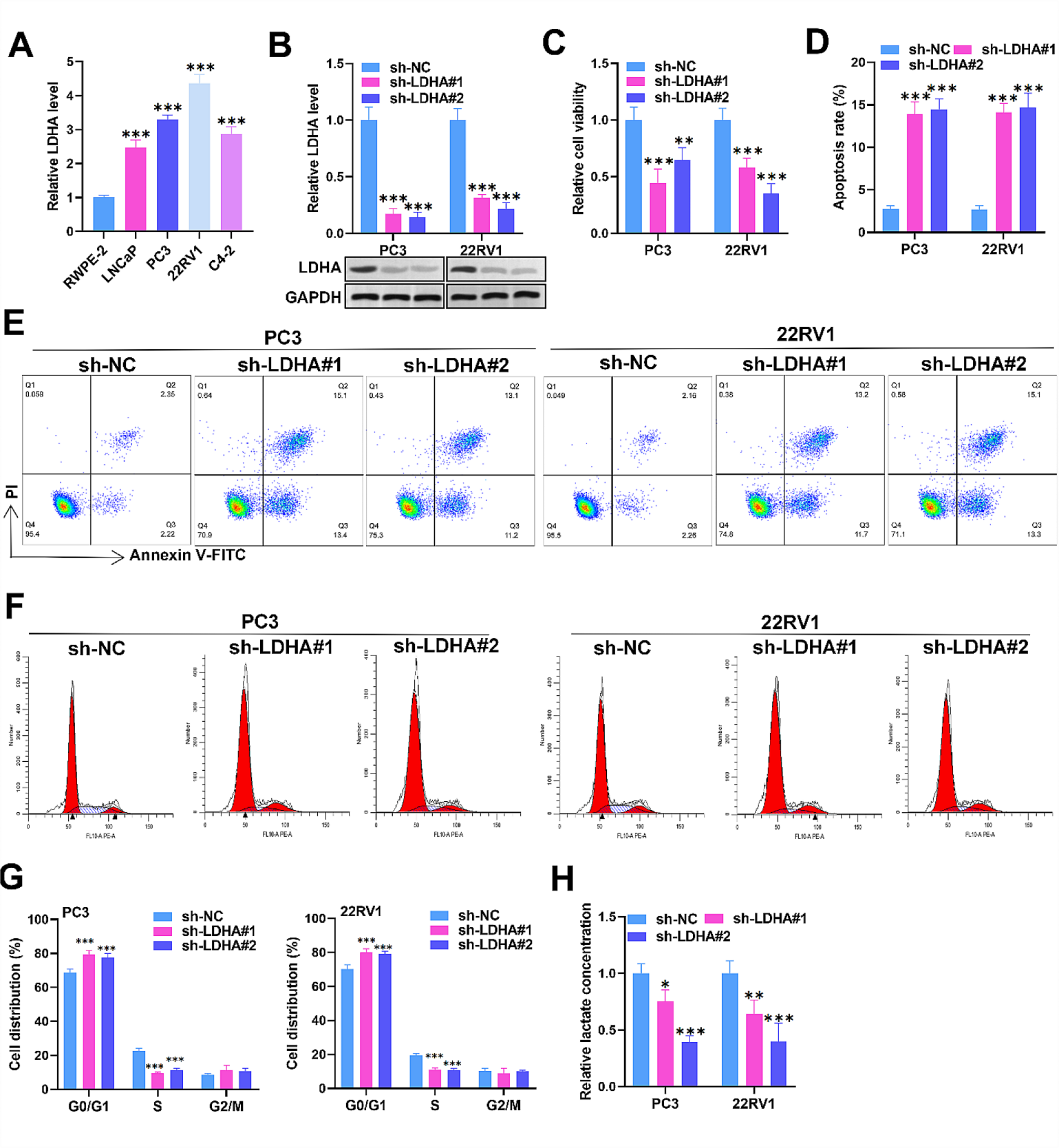
LDHA silencing increases apoptosis, induces cell cycle arrest, and suppresses glycolysis in PCa cells. (**A**) RT‒qPCR analysis of LDHA expression in PCa cells. (**B**) RT‒qPCR and western blot analysis of the transfection efficiency of sh-LDHA#1/2 in PC3 and 22RV1 cells. (**C**) Cell viability was evaluated by a CCK-8 assay in PC3 and 22RV1 cells transfected with sh-NC, sh-LDHA#1, or sh-LDHA#2. (**D-G**) Cell apoptosis and cell cycle progression were assessed via flow cytometry in different cells. (**H**) The lactate concentration in the cells was determined. The data is representative of at least three independent experiments. ^*^p < 0.05, ^**^p < 0.01, ^***^p < 0.001

We then evaluated the biological function of LDHA in PC cells. We first found that LDHA was strongly expressed in LNCaP, PC3, 22RV1, and C4-2 cells (Fig. 4A). Then, LDHA was silenced in PC3 and 22RV1 cells by sh-LDHA transfection. Both RT‒qPCR and western blot confirmed the successful downregulation of LDHA (Fig. 4B). Using a CCK-8 kit and flow cytometry, we found that LDHA depletion markedly restrained cell viability and enhanced cell apoptosis (Fig. 4C-E). Furthermore, LDHA depletion decreased the S phase ratio and elevated the G0/G1 phase ratio in PC3 and 22RV1 cells (Fig. 4F-G). In addition, we observed decreased lactate production in PC3 and 22RV1 cells after sh-LDHA transfection (Fig. 4H). Overall, LDHA silencing increases apoptosis, induces cell cycle arrest, and represses glycolysis in PCa cells.

### LDHB overexpression promotes apoptosis, induces G0/G1 cell cycle arrest, and reduces glycolysis in PCa cells

**Fig. 5:**
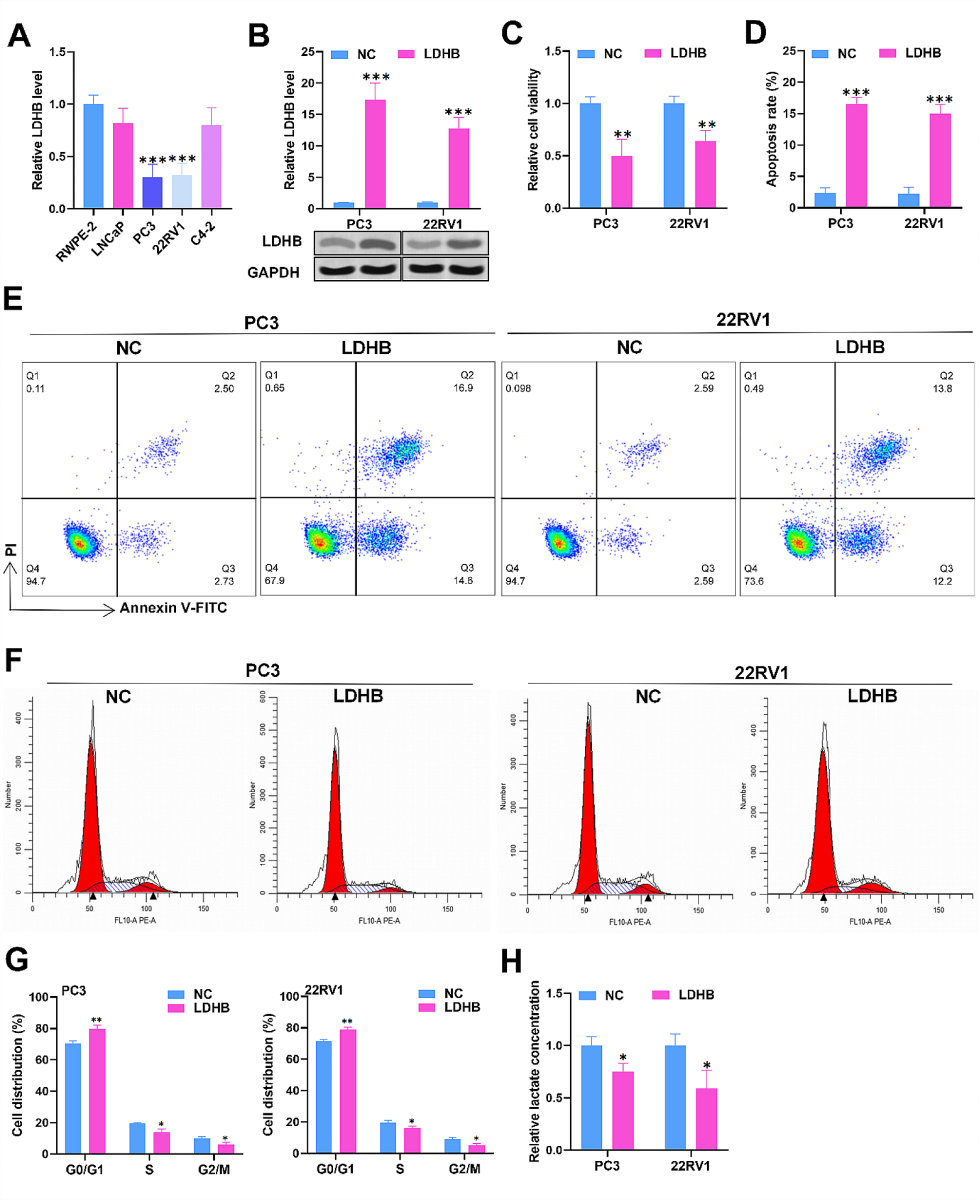
LDHB overexpression facilitates apoptosis, induces G0/G1 cell cycle arrest, and reduces glycolysis in PCa cells. (**A**) RT‒qPCR analysis of LDHB expression in PCa cells. (**B**) RT‒qPCR and western blot analysis of the transfection efficiency of pcDNA3.1-LDHB in PC3 and 22RV1 cells. (**C**) Cell viability was determined by a CCK-8 assay in PC3 and 22RV1 cells transfected with pcDNA3.1-NC or pcDNA3.1-LDHB. (**D-G**) Cell apoptosis and the cell cycle were assessed via flow cytometry analysis in different cells. (**H**) The lactate concentration was measured in the cells. The data is representative of at least three independent experiments. ^*^p < 0.05, ^**^p < 0.01, ^***^p < 0.001

The biological function of LDHB in PC cells was further determined. RT‒qPCR revealed notable downregulation of LDHB in PC3 and 22RV1 cells (Fig. 5A). LDHB was subsequently overexpressed in PC3 and 22RV1 cells after pcDNA3.1-LDHB transfection (Fig. 5B). Next, we proved that LDHB overexpression significantly suppressed cell viability and elevated the cell apoptotic ratio (Fig. 5C-E). LDHB upregulation induced cell cycle arrest at the G0/G1 phase in PC3 and 22RV1 cells (Fig. 5F-G). Lactate production was also decreased by LDHB overexpression (Fig. 5H). Overall, LDHB overexpression promotes apoptosis, induces cell cycle arrest, and suppresses glycolysis in PCa cells.

### STAT1 is positively regulated by FGF1/2, promotes LDHA expression, and decreases LDHB expression

**Fig. 6:**
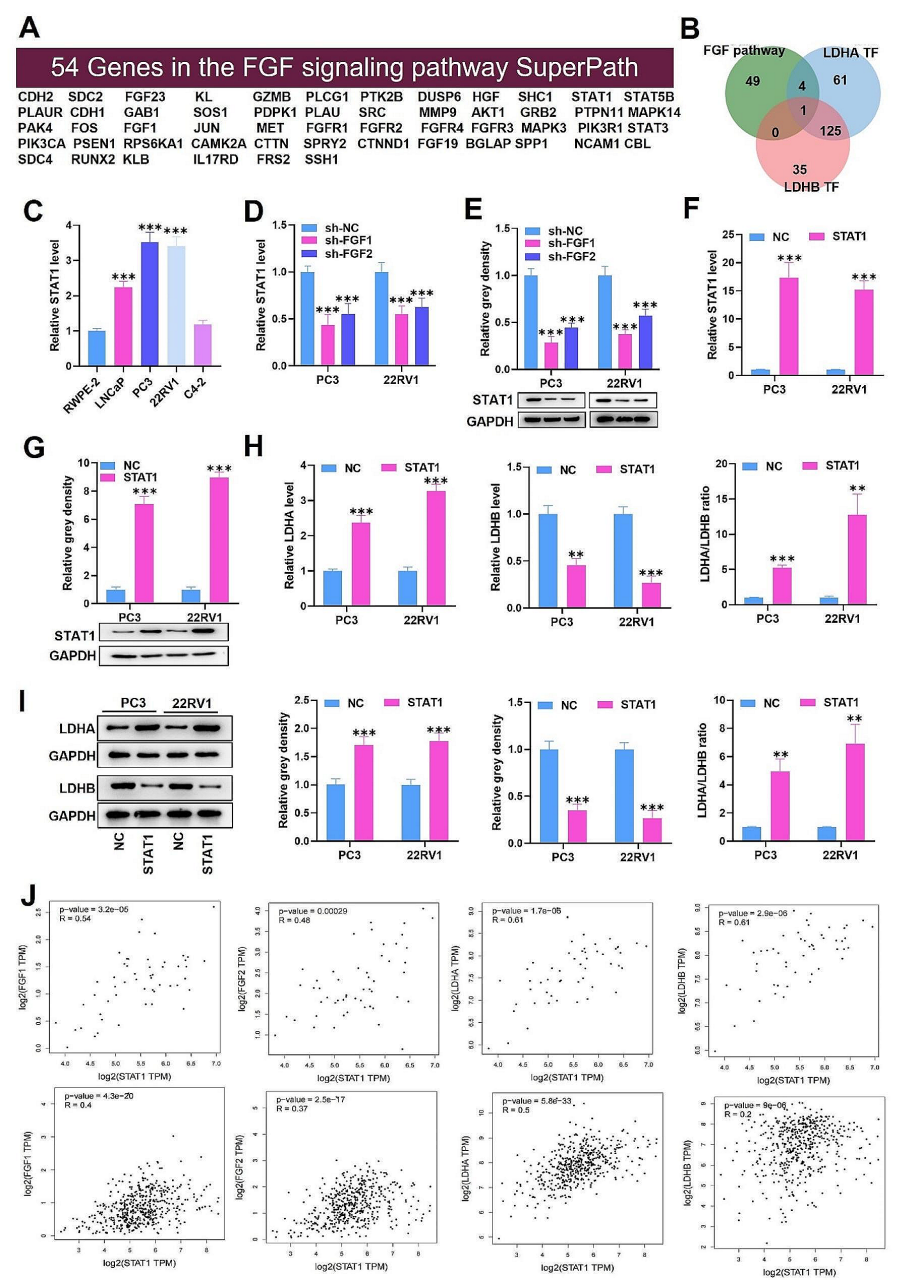
STAT1 is positively regulated by FGF1/2, promotes LDHA expression, and decreases LDHB expression. (**A**) The PathCards database was utilized to predict the genes related to the FGF signaling pathway. (**B**) Venn diagram showing a unique common intersection between FGF signaling pathway-related genes, LDHA upstream transcription factors, and LDHB upstream transcription factors. (**C**) RT‒qPCR outcomes of STAT1 expression in PCa cells. (**D-E**) RT‒qPCR and western blot analysis of STAT1 expression in cells transfected with sh-FGF1 or sh-FGF2. (**F-G**) RT‒qPCR and western blot analysis of the transfection efficiency of pcDNA3.1-STAT1 in cells. (**H-I**) RT‒qPCR and western blot analysis of LDHA expression, LDHB expression, and the LDHA/LDHB ratio in STAT1-overexpressing cells. (**J**) Spearman correlation analysis of STAT1 with LDHA, LDHB, FGF1, or FGF2 in normal prostate tissues (first row) and PRAD tissues (second row). The data is representative of at least three independent experiments. ^**^p < 0.01, ^***^p < 0.001

We further investigated the molecular mechanism by which FGF1/2 regulated LDHA and LDHB. Using the PathCards database (https://pathcards.genecards.org/Card/fgf_signaling_pathway?queryString=FGF), we obtained 54 genes related to the FGF pathway (Fig. 6A). Then, through the hTFtarget database (http://bioinfo.life.hust.edu.cn/hTFtarget#!/), we obtained the upstream transcription factors for LDHA and LDHB. By intersecting the genes selected above, we obtained only the gene signal transducer and activator of transcription 1 (STAT1) (Fig. 6B). We then measured STAT1 expression in PCa cells, and we observed notable upregulation of STAT1 in LNCaP, PC3, and 22RV1 cells (Fig. 6C). The STAT1 expression in PCa was also explored based on the TCGA database in USCS Xena database. We found that STAT1 showed higher levels in metastatic PCa (Fig. S1C). Then, we explored the impacts of FGF1/2 on STAT1 expression in cells. STAT1 mRNA and protein levels were obviously decreased by FGF1/2 depletion, suggesting that FGF1/2 can positively modulate STAT1 (Fig. 6D-E). Accordingly, we assumed that STAT1 regulated LDHA and LDHB in PCa. We then overexpressed STAT1 in PC3 and 22RV1 cells by pcDNA3.1-STAT1 transfection (Fig. 6F-G). We found that STAT1 overexpression notably elevated LDHA expression and reduced LDHB expression at the mRNA and protein levels (Fig. 6H-I). The LDHA/LDHB ratio was also promoted by STAT1 overexpression (Fig. 6H-I). Based on the Spearman correlation analysis in GEPIA, we identified a significant correlation between STAT1 and LDHA/LDHB/FGF1/FGF2 in tumor tissues but not in normal tissues (Fig. 6J). Overall, this data suggests that STAT1 is positively modulated by FGF1/2 and promotes LDHA expression and decreases LDHB expression.

### STAT1 is a transcription factor for LDHA and LDHB and promotes glycolysis in PCa cells

**Fig. 7:**
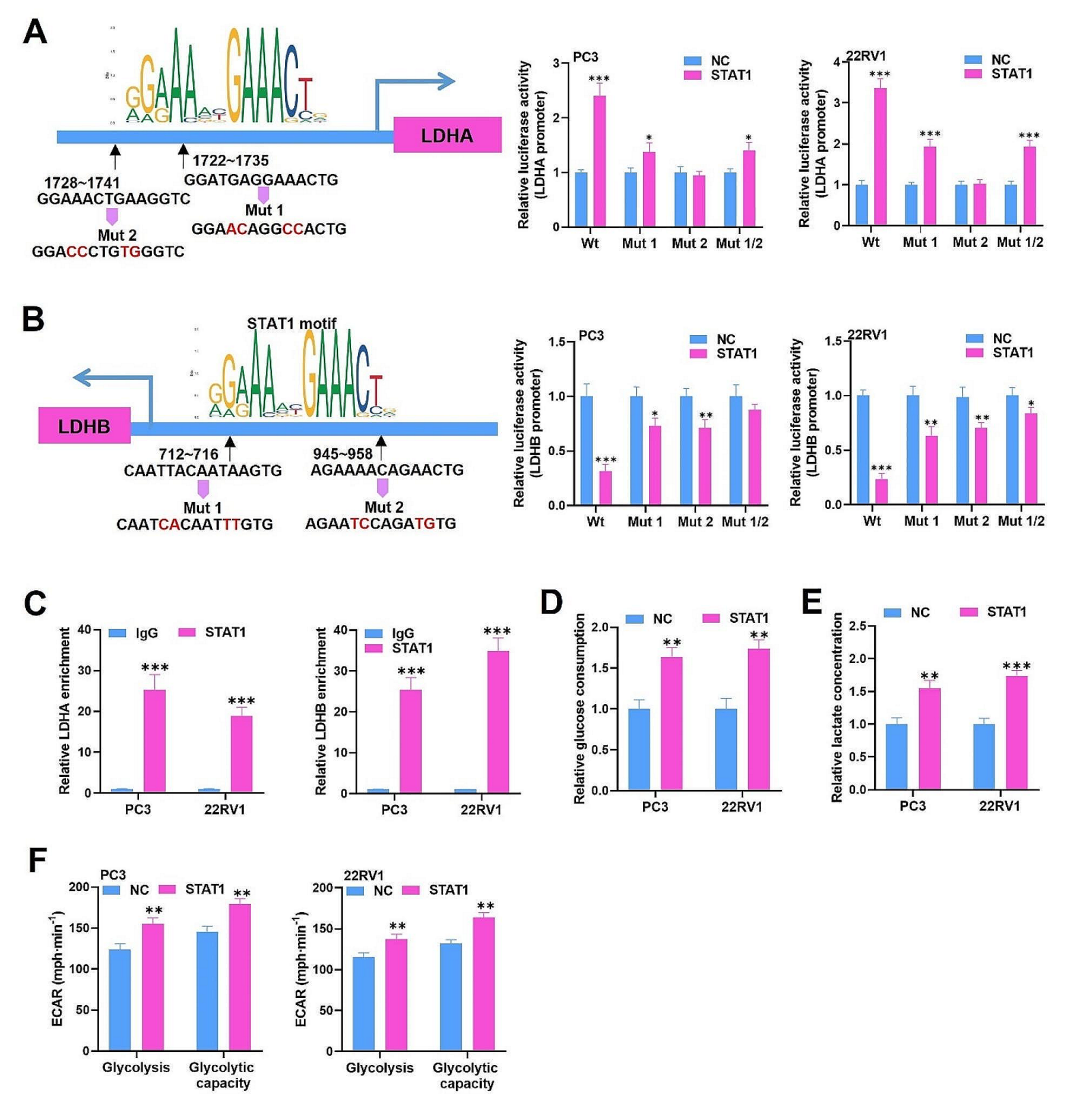
STAT1 is a transcription factor for LDHA and LDHB and promotes glycolysis in PCa cells. (**A-B**) The JASPAR database was utilized for predicting the motif of STAT1 and the binding sites of STAT1 and the LDHA (**a**)/LDHB (**b**) promoter. A luciferase reporter assay was implemented to verify the interaction between STAT1 and the LDHA (**a**)/LDHB (**b**) promoter. (**C**) A ChIP assay was performed to further verify the interaction between STAT1 and the LDHA/LDHB promoter. (**D-E**) Glucose consumption and lactate concentration were determined in STAT1-overexpressing cells. (**F**) The ECAR test also confirmed the glycolytic and glycolytic capacity of the cells. The data is representative of at least three independent experiments. ^*^p < 0.05, ^**^p < 0.01, ^***^p < 0.001

We then investigated whether STAT1 is involved in the regulation of LDHA or LDHB and related metabolic processes. According to the JASPAR database (https://jaspar.genereg.net/), we predicted the binding sites between STAT1 and LDHA or LDHB. The binding sites and the DNA motif of STAT1 were shown in Fig. 7A. To confirm these binding sites, luciferase reporter assays were performed. We constructed two mutants (Mut 1 and Mut 2) by mutating each promoter, and the cells were co-transfected with pcDNA3.1-STAT1 with Wt, Mut 1, Mut 2, or both the Mut 1 and Mut 2 plasmids of the LDHA promoter. Our results illustrated that, compared with the controls, the Wt luciferase activity was markedly elevated by STAT1 overexpression, suggesting that LDHA was the direct target of STAT1. We further observed that the activities of Mut 1 and Mut1 + Mut2 in STAT1-overexpressing cells were significantly higher than those in the control, whereas there was almost no difference between Mut 2 activity and the control. Taken together, these results indicated that STAT1 targeted the LDHA promoter mostly through binding to site 2 (1728 ∼ 1741) rather than site 1 (1722 ∼ 1735) (Fig. 7A). Similarly, luciferase reporter assays were carried out to verify the interaction between STAT1 and LDHB. The outcomes illustrated that the Wt luciferase activity of the LDHB promoter was notably restrained by STAT1 upregulation. After mutation of any of the sites, the luciferase activity decreased. However, the combination of Mut 1 and Mut 2 blocked the decrease in luciferase activity (Fig. 7B). These results suggested that STAT1 targets the LDHB promoter through binding to site 1 and site 2. Additionally, ChIP assays demonstrated that STAT1 was highly bound to the LDHA or LDHB promoter (Fig. 7C). After verifying the interaction between STAT1 and LDHA/LDHB, we explored the impact of STAT1 on glycolysis. STAT1 overexpression elevated glucose consumption, lactate production, glycolysis, and glycolytic capacity in PC3 and 22RV1 cells, suggesting that STAT1 promotes glycolysis in PCa (Fig. 7D-F). Overall, we proved that STAT1 is a transcription factor for LDHA and LDHB and promotes glycolysis in PCa cells.

### FGF pathway inhibitor suppresses tumor growth and reduces the lactate concentration in vivo

**Fig. 8:**
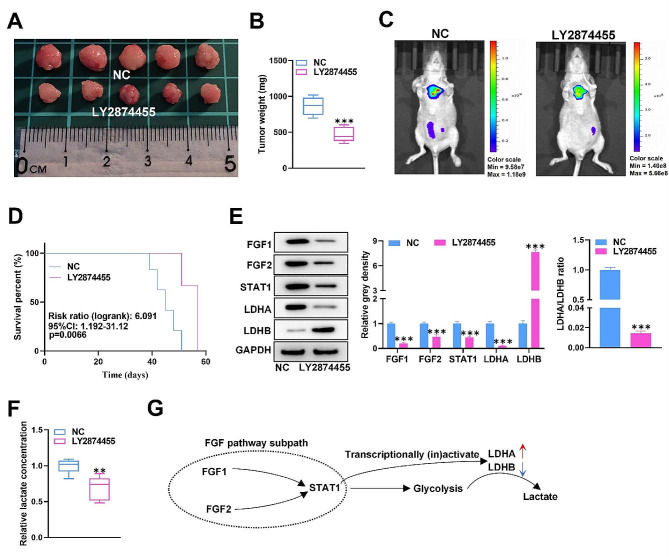
FGF pathway inhibitor suppresses tumor growth and reduces the lactate concentration in vivo. (**A-D**) Images of the tumors, tumor weights, bioluminescence signals, and survival rates of the mice in the NC group and LY2874455 group were recorded and measured. (**E**) Western blot of FGF1, FGF2, STAT1, LDHA, and LDHB in tumor tissues from the NC group and LY2874455 group. (**F**) The lactate concentration in tumor tissues was tested. (**G**) Graphical abstract of the study. ^**^p < 0.01, ^***^p < 0.001

Finally, we implemented animal experiments to verify the tumorigenic function of the FGF pathway in PCa. A xenograft mouse model of PCa was established by implanting PC3 cells into nude mice. Another group of model mice was treated with LY2874455 (an FGF/FGFR inhibitor). The experimental results illustrated that, in comparison with control mice, LY2874455-treated mice had a lower tumor growth rate, as evidenced by a lower tumor weight and less bioluminescence (Fig. 8A-C). In addition, we recorded the death time of the mice and found that LY2874455 treatment notably improved the survival rate of the tumor-bearing mice (Fig. 8D). Western blot analysis illustrated that LY2874455 treatment restrained FGF1, FGF2, STAT1, and LDHA levels but increased LDHB levels in tumor tissues. Moreover, the LDHA/LDHB ratio was also suppressed by LY2874455 (Fig. 8E). We subsequently utilized a lactic acid test kit and found that the lactate concentration in tumor tissues was notably reduced by LY2874455 (Fig. 8F). Thus, we proved that inhibiting the FGF pathway suppresses tumor growth and reduces the lactate concentration in vivo.

## Discussion

PCa is a common malignant tumor in men. Metabolic reprogramming from oxidative phosphorylation to aerobic glycolysis is a typical feature of tumor cells [43]. An increase in glycolysis is manifested by an increase in glucose consumption and lactate production and can provide energy to support tumor growth [44]. Therefore, targeting glycolysis provides an effective method for controlling tumor growth. LDH is an enzyme that can catalyze the reversible conversion of pyruvate to lactate [45]. LDHA promotes the conversion of pyruvate to lactate, so it is the key enzyme that promotes glycolysis. LDHB is converted to promote oxidative phosphorylation. The specific catalytic direction depends on the LDHA/LDHB ratio [46]. Bioinformatics analysis revealed that LDHA was upregulated in PRAD patients, and upregulated LDHA was associated with a low survival rate, high Gleason score, and high nodule metastasis status in PRAD patients. In contrast, LDHB expression was downregulated in PRAD patients, and this change was accompanied by decreased Gleason scores. In our study, we found that knocking down LDHA markedly prevented cell proliferation and cell cycle progression. LDHB overexpression also exerted similar effects on PCa cells. In addition, we observed a decrease in the lactate concentration in LDHA-silenced or LDHB-overexpressing cells. Therefore, we confirmed that LDHA promoted malignant phenotypes and aerobic glycolysis in PCa cells, while LDHB had the opposite effects. LDHA is overexpressed in different cancer cells and is considered a biomarker for multiple malignant tumors and is closely related to poor prognosis [47]. For example, LDHA increases glycolysis and proliferation in thyroid cancer [15]. Elevated LDHA enzyme activity is conducive to promoting glycolysis and proliferation in renal cell carcinoma cells [48]. In addition, Liu et al. demonstrated that LDHA enhances the malignant progression of PCa by promoting glycolysis, and LDHB plays the opposite role in PCa cells [19]. Our results also revealed the contrary functions of LDHA and LDHB in PCa development, which are in line with these reports.

FGF signaling is important for the malignant progression of tumors [21]. FGF1 and FGF2 belong to the FGF family and are key participants in the proliferation and differentiation of various cells and tissues [21]. It has been reported that FGF1 upregulation stimulates angiogenesis and reduces overall survival in patients with ovarian cancer [49]. It can also enhance resistance to etoposide and cisplatin in ovarian cancer cells [50]. FGF1 enhances the tumorigenicity and proliferation of colorectal cancer cells [51]. Moreover, FGF2 overexpression is conducive to bladder cancer cell migration and angiogenesis [52, 53]. FGF2 can activate the ERK1/2 pathway and elevate MYC levels to reduce sensitivity to endocrine therapy and facilitate tumor growth in patients with breast cancer [54]. Mechanistic research has established a connection between FGF1/2 and PCa. Udayakumar et al. revealed that FGF1 can induce the expression of matrix metalloproteinases in PCa cells, thus participating in malignant development of PCa [34]. Polnaszek et al. suggested that FGF2 ablation notably suppresses tumor growth in poorly differentiated PCa, reduces distant metastasis, and extends the life of mice [55]. Our study demonstrated that FGF1/2 are highly expressed in PCa cells. FGF1/2 positively modulates LDHA expression and negatively modulates LDHB expression. Accordingly, the LDHA/LDHB ratio was positively regulated by FGF1/2. Evidence has revealed that an elevated LDHA/LDHB ratio is closely related to the clinical outcomes of PCa patients [46]. Furthermore, our study showed that FGF1/2 deficiency markedly restrained PCa cell proliferation, the cell cycle, glucose consumption, the lactate concentration, and glycolytic capacity. Therefore, we concluded that FGF1/2 enhances glycolysis in PCa cells by promoting LDHA and inhibiting LDHB.

The STAT family of transcription factors plays vital roles in modulating cell processes, including proliferation, apoptosis and angiogenesis [56]. They translocate from the cytoplasm to the nucleus to function as transcriptional inhibitors or activators under the stimulation of growth factors [57]. STAT1 is a member of the STAT family. Previous studies have revealed that STAT1 has both carcinogenic and tumor suppressive functions in different cancers, which may depend on the background of the cancer cells [58–61]. In PCa, STAT1 is found to be overexpressed and accelerate cell proliferation, migration and the cell cycle [62]. STAT1, a common transcription factor, functions in both facilitating and suppressing the transcription of downstream genes [63]. For example, STAT1 transcriptionally suppresses miR-181a expression to restrain colorectal cancer cell growth [64]. STAT1 transcriptionally activates ERα expression to accelerate breast cancer development [60]. Herein, we discovered that STAT1 expression was positively modulated by FGF1/2 in PCa cells. Through bioinformatics tools, STAT1 may serve as a transcription factor for LDHA and LDHB. Mechanistic assays confirmed that STAT1 can bind to the promoter regions of LDHA/LDHB. Notably, STAT1 transcriptionally activated LDHA but transcriptionally inhibited LDHB. Overexpression of STAT1 elevated LDHA expression in PCa cells and restrained LDHB expression. Additionally, the LDHA/LDHB ratio was also increased by STAT1 overexpression. It has been reported that STAT1 is the crucial driver of increased glycolysis in myeloma cells [65]. Pitroda et al. suggested that STAT1 participates in the transcriptional regulation of the Warburg effect in cancers [66]. Our study demonstrated that STAT1 upregulation increased glucose consumption and the lactate concentration and enhanced glycolysis in PCa cells.

The involvement of the FGF pathway in PCa occurrence suggests that blocking this pathway can be used for the treatment of PCa patients. Therefore, we implemented xenograft experiments to further validate our findings. The results demonstrated that LY2874455 (an FGF inhibitor) treatment notably suppressed tumor growth, improved the survival rate, and reduced the LDHA/LDHB ratio and serum lactate concentration. This evidence further confirmed our research findings.

In conclusion, our findings demonstrated that the FGF pathway facilitates glycolysis by altering the LDHA/LDHB ratio through the activation of LDHA and suppression of LDHB in a STAT1-dependent manner in PCa cells, which enables PCa cells to fully utilize glycolysis to meet cell proliferation needs (Fig. 8G). The findings of our study provide new potential therapeutic targets for PCa treatment.

### Electronic supplementary material

Below is the link to the electronic supplementary material.

Supplementary Material 1

## Data Availability

The datasets generated during and/or analyzed during the current study are available from the corresponding author upon reasonable request.
