# Supplementary material for: Fibroblast growth factor pathway promotes glycolysis by activating LDHA and suppressing LDHB in a STAT1-dependent manner in prostate cancer
Source: J Transl Med. 2024 May 19;22:474. doi: 10.1186/s12967-024-05193-9 (PMC11103983; doi:10.1186/s12967-024-05193-9)
Supplement: Supplementary file 1 — Supplementary Material 1 [file 12967_2024_5193_MOESM1_ESM.docx]

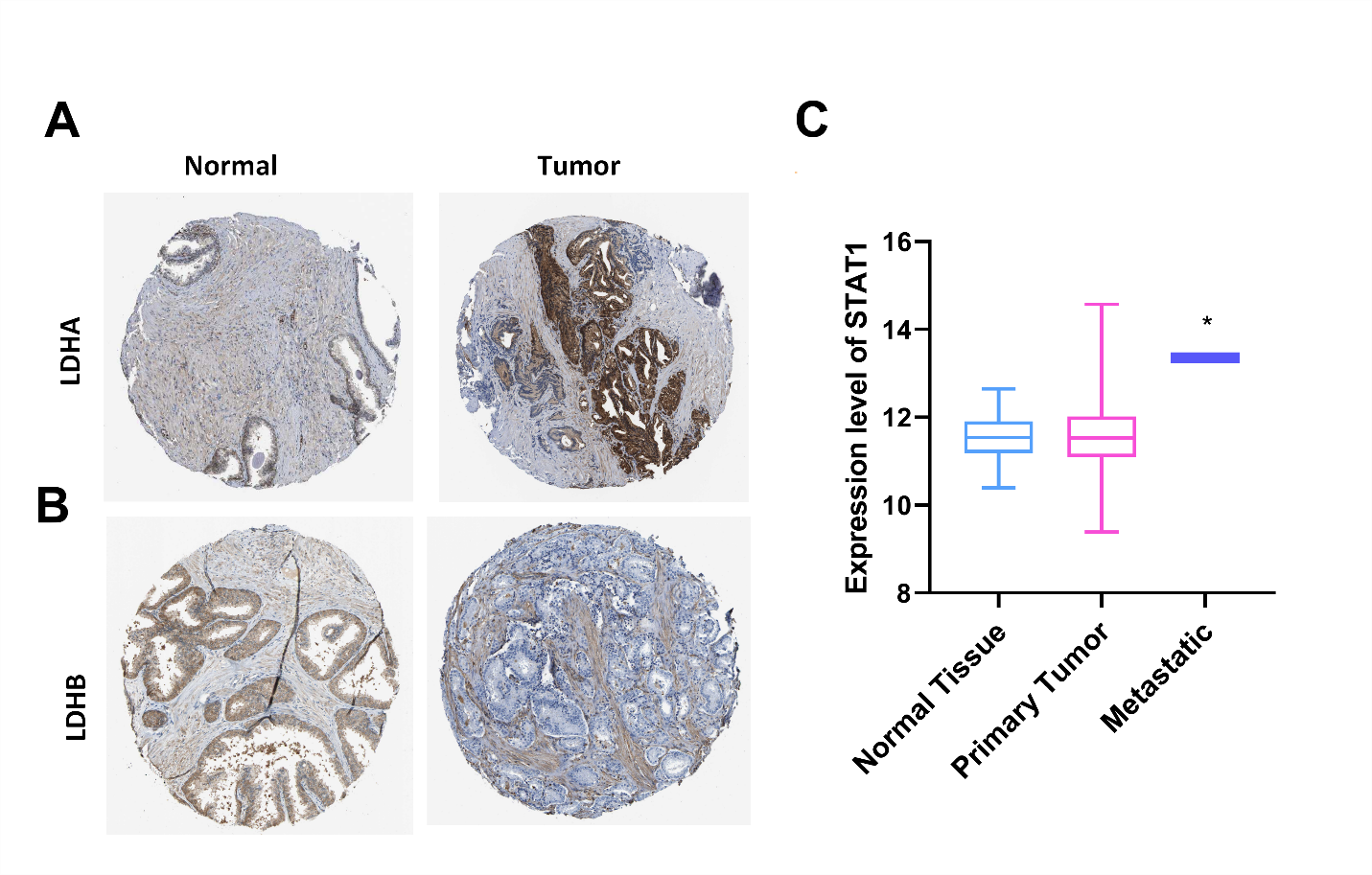


**Supplementary figure S1. Expression of LDHA, LDHB proteins, and STAT1 mRNA in PCa.** THE HUMAN PROTEIN ATLAS (HPA) database (https://www.proteinatlas.org/) was used to explore the protein expression of (A) LDHA and (B) LDHB in prostate adenocarcinoma samples and normal samples via immunohistochemistry. (C) The expression of STAT1 in normal tissue, primary PCa samples, and metastatic PCa samples was explored using the TCGA data downloaded from the UCSC Xena database. ^*^*p*<0.05.
